# Reliability and reproducibility of spectral and time domain optical coherence tomography images before and after correction for patients with age-related macular degeneration

**DOI:** 10.12688/f1000research.2-131.v2

**Published:** 2015-03-05

**Authors:** Mohammad A. Sadiq, Aymen Rashid, Roomasa Channa, Elham Hatef, Diana V Do, Quan Dong Nguyen, Yasir J Sepah

**Affiliations:** 1 Ocular Imaging Research and Reading Center (OIRRC), Stanley M Truhlsen Eye Institute, University of Nebraska Medical Center, Omaha, NE, 68198, USA

**Keywords:** age related macular degeneration, neovascularization, optical coherence tomography, spectral domain, time domain

## Abstract

**Purpose:** To evaluate the reproducibility and reliability of optical coherence tomography scans obtained using the time domain (TD-OCT) Stratus ^TM^ OCT, and the Spectral Domain (SD-OCT) Spectralis ^TM^ and Cirrus ^TM^ OCT devices before and after manual correction in eyes with either Neovascular (NV-AMD) or Non-Neovascular (NNV-AMD) age-related macular degeneration.

**Design:** Prospective observational study.

**Methods:**

Setting: University-based retina practice.

Patients: Thirty-six patients (50 eyes) with NV-AMD or NNV-AMD.

Procedure: OCT scans were taken simultaneously using one TD-OCT and two SD-OCT devices.

Main Outcome Measures: Macular thickness measurements were assessed before and after correction of the algorithm by constructing Bland-Altman plots for agreement and calculating intraclass correlation coefficients (ICCs) and coefficients of repeatability (COR) to evaluate intraclass repeatability.

**Results: **Spectralis had the highest number of images needing manual correction.  All machines had high ICCs, with Spectralis having the highest.  Also, Bland-Altman plots indicated that there was low agreement between Cirrus™ and Stratus™, Spectralis™ and Stratus™, while there was good agreement between the Cirrus™ and Spectralis™.  The CORs were lowest for Spectralis ^TM ^and similar and higher for Cirrus ^TM ^and Stratus ^TM^.  Agreement, CORs, and ICCs generally improved after manual correction, but only minimally.

**Conclusion: **Agreement is low between devices, except between both SD-OCT machines.  Manual correction tends to improve results.

## Introduction

Optical Coherence Tomography (OCT) is a non-invasive imaging modality that allows acquisition of cross-sectional images of the retina. OCT is useful in monitoring and evaluating retinal thickness in many retinal disorders. One example is Age-related Macular Degeneration (AMD), a progressive, blinding disease that is mostly non-neovascular (NNV-AMD) but can be associated with choroidal neovascularization (NV-AMD). Currently, OCT is also being employed as an outcome measure in many multicenter clinical trials of AMD with Time Domain OCT (TD-OCT) device being the most common ^1, 2^.

As this technology is increasingly being utilized by many ophthalmologists to evaluate and monitor patients and guide treatment decisions ^2^, it is important to understand the reliability and accuracy of thickness measurements obtained with various devices currently available. Recently, studies have shown that in patients with AMD, there is a high frequency of errors in automated retinal thickness measurements due to incorrect segmentation of the retina in the TD-OCT machine specifically in NV-AMD ^2, 3^. Using an Spectral Domain OCT (SD-OCT) device Menke *et al.* found that NNV-AMD had fewer errors than NV-AMD, mostly due to the pathology of the disease resulting in retinal pigment epithelial (RPE) layer changes ^4^.

Manual correction of the algorithm is an option in newer generations of the review software and as more OCT devices are coming to the market, it is important to understand the clinical importance of manual correction of OCT algorithms and the agreement of thickness measurements from different machines before and after correction. In our study, we evaluated the intra-session repeatability and agreement in retinal thickness measurements for patients with NV-AMD and NNV-AMD before and after manual correction using three different OCT devices: Stratus™ TD-OCT and two SD-OCTs, Spectralis™ and Cirrus™.

## Methods

Institutional Review Board (IRB)/Ethics Committee approval was obtained and HIPAA guidelines were followed for the study. Informed consent was obtained from study subjects.

## Patients and scanning

Patients with confirmed diagnosis of AMD were enrolled in the study. Two senior retina specialists (QDN and DVD) made the diagnosis of AMD. Patients under treatment with intravitreal injections of anti-vascular endothelial growth factor (VEGF) agents were also allowed to participate in the study.

Patients were scanned twice by certified OCT operators on a TD-OCT device (Stratus™ OCT) and two SD-OCT devices (Spectralis™, and Cirrus™ OCT) machines in random order and with 5–10 minutes between each device. The same operator performed all the scans on any given patient. Scans on a single device were performed consecutively and 5 minutes apart from each other.

## Optical Coherence Tomography

One TD-OCT machine, Stratus™ (software version 4), and two SD-OCT machines, Spectralis™ (software version 5.0 I and Cirrus™ (software version 5.0.0.326) were used. Stratus™ is a TD-OCT machine that uses a super luminescent diode with a wavelength of 820 nm. It provides an axial resolution of 10µm and image acquisition speed of 400 A-scans/second. Using the Stratus™, two fast macular thickness maps (FMTP) were acquired from each eye. The FMTM is created through acquiring six radial B-scans, each consisting of 512 A-scans, and at an angle of 30° from each other with the point of intersection centered on the fovea.

Spectralis™ uses a super luminescent diode with a wavelength of 870 nm. It provides axial resolution of 4µm and image acquisition speeds of up to 40,000 A-scans per second. Two volume scans were acquired from each eye using a raster scan of 19 lines covering 20×15 ^o^ of the fundus. Using the TruTrack™ functionality of the Spectralis™ OCT, each line was averaged 15 times or more. Cirrus™ HD-OCT also uses a super luminescent diode with a wavelength of 840 nm. It provides images with an axial resolution of 5µm and acquisition speeds of 27,000 A-scans per second. We acquired two 512×128 macular cube scans (128 B-scans and 512 A-scans, covering a retinal area of 6.0×6.0 mm) from each eye.

## Error determination, manual correction, and exclusion of scans

Scans from each of the three devices were reviewed at the Ocular Imaging Research and Reading Center at the Stanley M. Truhlsen Eye Institute by two independent graders. Segmentation errors due to incorrect identification of inner and outer retinal boundaries by automated algorithms in the Spectralis™ and Cirrus™ devices were identified and manually corrected by these graders. Stratus™ images could not be corrected due to the lack of editing capabilities in the operating system provided with the machine at the time of conducting the study. Only 5 patients required corrections and were excluded from the analysis. The proprietary software identifies retinal boundaries for measurement of retinal thickness that are specific to each device. Meanwhile each device identifies the inner limiting membrane (ILM) as the inner boundary of retina, identification of the outer boundary is different for each device. Stratus™ identifies the junction between the inner and outer segments of photoreceptors (IS/OS) as the outer boundary, Spectralis™ identifies the posterior border of the retinal pigment epithelium (RPE), and Cirrus™ identifies the inner border of the RPE as the outer retinal boundary.

Whenever the foveal center could be identified, grids were repositioned for scans with off-center positioning of the ETDRS grid. However, in some cases, morphological changes associated with the advanced disease made identification of the foveal center unreliable. Adjustment of grid position was not possible for Stratus™ OCT. Scans were excluded from analysis only if identification of retinal layers and determination of the retinal thickness was not possible. OCT scans from which extraction of thickness data for the central 1mm sub-field was not reliable, due to missing data in the image or the scan being out of range, were also excluded from analysis.

The retinal thickness measurements of the nine standard ETDRS subfields ( Appendix A illustrates the nine-subfield abbreviations) were recorded from each device before and after correcting the errors in the scans algorithm.

## Statistical analysis

No formal sample size calculation was performed before the conduct of the study. Bland-Altman plots were constructed to determine agreement between devices; both 95% confidence intervals and limits of agreements were calculated. Reproducibility of measurements was determined by calculating the coefficients of repeatability (COR) for each machine. Intraclass correlation coefficients (ICCs) were used to determine the reproducibility for each device. Statistical significance of difference in thickness before and after correction of images across devices was determined via student’s t-test with α = 0.05 with Bonferroni correction for multiple comparisons. STATA version 10 and Microsoft Excel 2007 were used for data management and analysis. The statistical analysis was performed before and after any manual corrections were made to the algorithm errors described above.

## Results

Fifty eyes from 36 patients were included in the study; 29 eyes had NV-AMD and 21 eyes had NNV-AMD. The mean age of the study subjects was 76.6 years.

## Exclusion and corrections

### Stratus™

Scans from four eyes could not be recovered from the database and scans from three eyes had algorithm errors with incorrect identification of retinal boundaries and were excluded from analysis. Scans were not corrected for off-center positioning of the scan as moving the ETDRS grid was not possible with the available software version.

### Cirrus™

Scans in six eyes scanned first and eight eyes scanned second were corrected either for off-center fixation of the eye or for incorrect automated identification of retinal boundaries. The thickness measurements before and after correction were not statistically significant (P<.05) for any of the subfields and also when stratified by diagnosis.

### Spectralis™

**Figure 1.**
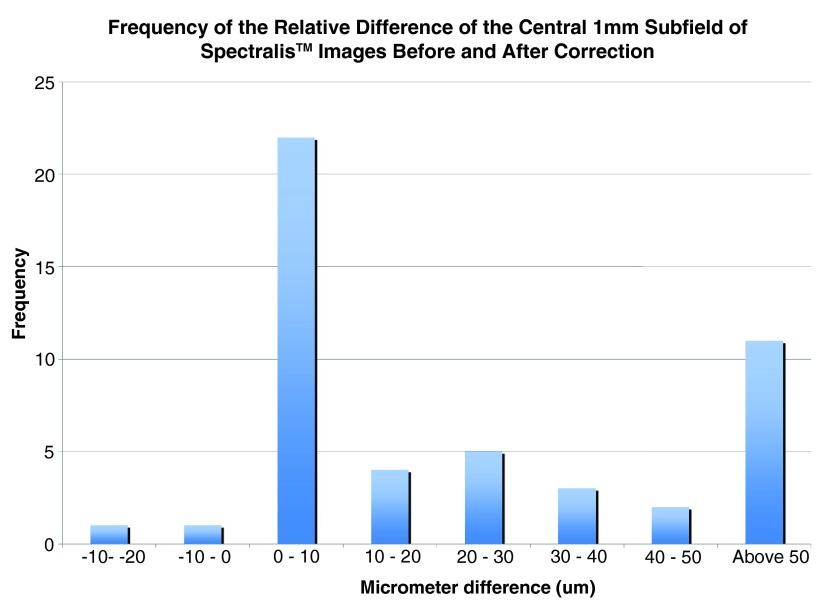
Frequency of the relative differences of the central 1mm subfield of Spectralis™ images before and after correction.

Thirty-three scans among the first set and 32 among the second set were corrected. The inner inferior subfield for NV-AMD was the only subfield that was statistically significant before and after correction. Figure 1 plots the frequency of the differences before and after correction for the central subfield for all scans. 77% of the differences were less than 48μm and 50% were less than 10μm.

## OCT characteristics

The mean (±SD) of the macular thickness of all of the subfields, including the central 1mm subfield (FTH) for Stratus™, Cirrus™, and Spectralis™ before and after manual correction of scans, stratified by diagnosis of NV-AMD and NNV-AMD, is shown in Table 1. For NV-AMD, the FTH values for central 1mm were 375µm (±129µm), 253µm (±74µm), 312µm (±110µm) for Spectralis™, Stratus™, and Cirrus™ respectively. After correction, the values were 335µm (±106µm) for Spectralis™ and 318µm (±110µm) for Cirrus™. On the other hand, the FTH values for NNV-AND in the central 1mm before correction were 298µm (87µm), 193µm (±32µm), and 229µm (±30µm) for Spectralis™, Stratus™, and Cirrus™ respectively. Spectralis™ was the only device to have a different FTH value of 248µm (±56µm) after correction. Overall, Spectralis™ had the highest retinal thickness values (range: 280 to 372µm), depending on the subfield. The retinal thickness measurements obtained via the Cirrus™ were slightly less (range: 230 to 320µm), while Stratus™ had the lowest values, ranging from 190 to 270µm. There were no significant (p<.05) differences between the mean FTH of the first and second scans for each of the three devices.

**Table 1.** A comparison of thickness measurements between two machines demonstrated that most values were significantly different (p<0.05). Spectralis™ vs. Cirrus™ before correction: for NV-AMD, T1, S1, and I2, and for NNV-AMD, C1, T1, N1, and I2. Spectralis™ vs. Cirrus™ after correction: for NV-AMD every field except S2, and I2 were not significant, and for NNV-AMD, the inner subfields were not significant. Spectralis™ vs. Stratus™ after correction: for NV-AMD, C1.

Mean ± standard deviation (µm)															
Subfield	All eyes					NV-AMD					NNV-AMD				
	Spectralis™		Stratus™	Cirrus™		Spectralis™		Stratus™	Cirrus™		Spectralis™		Stratus™	Cirrus™	
	Before	After	Before	Before	After	Before	After	Before	Before	After	Before	After	Before	Before	After
C1	343 ± 119	301 ± 98	229 ± 67	277 ± 94	281 ± 96	375 ± 129	335 ± 106	253 ± 74	312 ± 110	318 ± 110	298 ± 87	248 ± 56	193 ± 32	229 ± 30	229 ± 30
N1	348 ± 74	329 ± 70	267 ± 50	317 ± 57	319 ± 56	370 ± 81	351 ± 74	285 ± 54	336 ± 61	339 ± 61	319 ± 51	297 ± 51	239 ± 29	291 ± 36	291 ± 36
S1	346 ± 74	327 ± 66	260 ± 40	317 ± 71	316 ± 70	372 ± 84	349 ± 71	277 ± 40	342 ± 80	339 ± 80	312 ± 41	295 ± 44	234 ± 25	284 ± 36	284 ± 36
T1	345 ± 74	321 ± 59	250 ± 46	305 ± 65	305 ± 65	366 ± 83	337 ± 56	265 ± 51	325 ± 75	324 ± 75	318 ± 50	297 ± 57	229 ± 27	278 ± 33	278 ± 33
I1	347 ± 72	325 ± 70	256 ± 59	314 ± 66	314 ± 65	362 ± 78	340 ± 75	273 ± 63	329 ± 75	330 ± 74	327 ± 58	302 ± 58	230 ± 40	293 ± 44	293 ± 44
N2	307 ± 42	302 ± 44	250 ± 49	293 ± 46	292 ± 46	317 ± 49	312 ± 49	259 ± 60	302 ± 56	300 ± 58	293 ± 22	288 ± 31	237± 21	281 ± 21	281 ± 21
S2	297 ± 42	292 ± 42	223 ± 28	279 ± 46	278 ± 45	310 ± 47	303 ± 48	226 ± 34	291 ± 54	289 ± 55	282 ± 27	278 ± 28	218 ± 17	262 ± 21	262 ± 21
T2	284 ± 49	278 ± 46	220 ± 45	270 ± 66	269 ± 66	292 ± 54	286 ± 52	231 ± 53	283 ± 81	282 ± 82	273 ± 39	266 ± 36	205 ± 24	250 ± 25	250 ± 25
I2	292 ± 61	286 ± 63	233 ± 55	272 ± 42	270 ± 42	303 ± 75	298 ± 76	245 ± 64	278 ± 46	276 ± 46	276 ± 25	268 ± 28	214 ± 32	262 ± 34	262 ± 34

The central subfield ICC values for all three machines were very high at 99.6%, 97.2% and 96.4% before correction for Spectralis™, Stratus™, and Cirrus™ respectively, and 99.4%, and 97.4% after correction for Spectralis™ and Cirrus™. The ICC values were greater than 95% for all subfields and both diagnoses except the outer inferior field for NNV-AMD for Spectralis™. Stratus™ values ranged from 78.9% to 99.2% for NV-AMD and 94.7% to 99% for NNV-AMD, before and after correction, respectively. Cirrus™ values ranged from 88.5% to 99.9% and 99.1% to 99.8% for NV-AMD before and after correction, respectively. The values for NNV-AMD for Cirrus™ ranged from 99.3% to 99.9% and 71.4% to 99.7% before and after correction, respectively. Table 2 shows the ICC values between images for all three machines before and after correction, both combined and stratified by diagnosis. It should be noted that all of the machines had ICC values >90% for the central subfield while the Spectralis™ had no subfields less than 99% after correction. In the central subfield, Spectralis™ had a COR of 20µm NV-AMD which increased to 23µm; both Cirrus™ and Stratus™ had relatively larger CORs of 64µm (reduced to 49µm after correction) and 35µm, respectively. For NNV-AMD, the COR for the central subfield was 15µm for both Cirrus™ and Spectralis™, and was 24µm for Stratus™. After correction, the value decreased for Spectralis™ to 12µm and increased to 36µm for Cirrus™. The COR of all subfields for each device before and after correction of algorithms and stratification by disease are given in Table 3.

**Table 2.** Intraclass correlation coefficient percentages before and after correction.

ICC values (%)															
Subfield	(All eyes)					NV-AMD					NNV-AMD				
	Spectralis™		Stratus™	Cirrus™		Spectralis™		Stratus™	Cirrus™		Spectralis™		Stratus™	Cirrus™	
	Before	After	Before	Before	After	Before	After	Before	Before	After	Before	After	Before	Before	After
C1	99.6	99.4	97.2	96.4	97.4	99.8	99.6	98.5	97.7	98.8	99.7	99.6	95.9	98.4	92.4*
N1	99	99.5	87.6*	85.6*	84.7*	99.4	99.7	91.9*	99.9	90.6*	99.5	99.8	95.7	99.9	89.1*
S1	99.3	99.5	82.8*	93.8	90.7*	99.6	99.7	86*	95.8	95.3	99.5	99.6	96.3	98.7	88.9*
T1	98.2	99	96	95.6	90.4*	98.9	99.2	97.4	97.2	96.8	99.4	99.7	98.3	99.3	71.4*
I1	98.9	99	95.9	95.1	91.2*	99.4	99.7	98.3	96.6	93.6	99.3	99.1	94.7	99.4	99.4
N2	99.1	99.6	98.4	96.6	96	99.7	99.8	99.2	97.9	97.6	97.8	99.8	98.4	99.8	99.7
S2	99.6	99.5	71.2*	82*	90.5*	99.8	99.6	78.9*	88.5*	94*	99.7	99.8	97.2	99.5	99.4
T2	99.2	99.4	92.7	98.1	98.4	99.6	99.6	95.8	98.9	99.1	99.6	99.6	95.9	99.5	99.5
I2	98.3	99	94.9	95.8	95.6	99.8	99.4	96.8	97	97.2	90*	99.6	99	99.4	99.3

**Table 3.** Coefficient of the repeatability values before and after correction.

Coefficient of repeatability (μm)															
Subfield	All eyes					NV-AMD					NNV-AMD				
	Spectralis™		Stratus™	Cirrus™		Spectralis™		Stratus™	Cirrus™		Spectralis™		Stratus™	Cirrus™	
	Before	After	Before	Before	After	Before	After	Before	Before	After	Before	After	Before	Before	After
C1	18	20	31	50	44	20	23	35	64	49	15	12	24	15	36
N1	26	15	26	39	62	23	15	58	74	69	13	8	26	14	46
S1	16	12	49	49	58	20	13	62	63	64	10	10	18	15	48
T1	20	13	48	57	60	32	18	32	50	58	14	10	12	10	67
I1	20	18	32	38	53	22	14	31	48	67	18	22	33	13	13
N2	11	10	39	23	21	9	8	19	30	32	12	5	10	3	4
S2	6	7	42	48	35	6	9	53	62	45	6	4	11	6	6
T2	10	7	16	22	24	13	11	47	29	27	8	8	18	6	6
I2	19	15	35	26	25	9	19	44	32	31	28	6	12	10	10

Overall Spectralis™ had the lowest COR, with values ranging from 5–30µm. Cirrus™ and Stratus™ had similar values ranging from 5–70µm, even after correction. The COR for Cirrus™ increased by 15–40µm after correction for NNV-AMD. Also, Cirrus™ COR values were 10–30µm higher than Stratus™ values for both NV-AMD and NNV-AMD. Agreement between machines was poor, except between Spectralis™ and Cirrus™ after correction. Table 4– Table 5 show 95% confidence intervals and limits of agreement of the Bland-Altman plots between devices before and after manual correction.

**Table 4.** 95% Confidence Intervals for Bland-Altman Plots. **A**: Before correction. **B**: After correction.

A. Bland-Altman 95% confidence intervals before correction (µm)									
Subfield	All eyes			NV-AMD			NNV-AMD		
	Spectralis™ vs. Cirrus™	Cirrus™ vs. Stratus™	Spectralis™ vs. Stratus™	Spectralis™ vs. Cirrus™	Cirrus™ vs. Stratus™	Spectralis™ vs. Stratus™	Spectralis™ vs. Cirrus™	Cirrus™ vs. Stratus™	Spectralis™ vs. Stratus™
Central 1 mm	42, 89	37, 65	91, 149	33, 94	43, 84	87, 166	28, 109	18, 46	63, 156
N1	29, 63	44, 65	81, 118	25, 78	44, 76	79, 136	18, 60	36, 57	67, 110
S1	21, 49	44, 71	74, 109	16, 64	44, 86	75, 131	18, 37	34, 58	58, 90
T1	21, 43	41, 59	73, 99	18, 51	37, 64	74, 109	14, 41	37, 60	58, 99
I1	23, 43	41, 74	79, 111	18, 46	28, 81	71, 115	19, 49	45, 77	72, 123
N2	13, 24	40, 61	57, 81	11, 28	37, 60	52, 87	11, 22	37, 54	50, 86
S2	16, 27	42, 73	59, 93	13, 32	42, 91	57, 118	16, 25	38, 48	55, 71
T2	9, 17	35, 49	52, 65	7, 21	29, 51	48, 69	7, 17	38, 49	52, 64
I2	9, 33	28, 51	54, 67	7, 46	15, 47	47, 64	3, 26	40, 66	58, 79
Average Width	21.5	24	30.2	35.7	35.6	45	28	20.3	38.3
B. Bland-Altman 95% confidence intervals after correction (µm)									
Subfield	All eyes			NV-AMD			NNV-AMD		
	Spectralis™ vs. Cirrus™	Cirrus™ vs. Stratus™	Spectralis™ vs. Stratus™	Spectralis™ vs. Cirrus™	Cirrus™ vs. Stratus™	Spectralis™ vs. Stratus™	Spectralis™ vs. Cirrus™	Cirrus™ vs. Stratus™	Spectralis™ vs. Stratus™
Central 1 mm	8, 30	40, 70	55, 91	5, 29	48, 91	60, 110	(-1.7–42)	18, 46	27, 75
N1	11, 34	45, 64	63, 88	13, 32	45, 74	65, 92	(-2.6, 47)	36, 57	43, 95
S1	5, 29	43, 69	56, 87	(-.10, 38)	42, 83	56, 101	3, 26	34, 58	41, 78
T1	3, 21	44, 58	53, 79	.46, 26	44, 62	55, 89	(-1., 22)	37, 60	35, 76
I1	4, 17	41, 74	60, 86	(-.20, 19)	29, 81	55, 88	4, 22	44, 78	50, 100
N2	8, 20	39, 60	53, 74	5, 25	35, 68	46, 82	7, 16	37, 54	49, 78
S2	10, 26	41, 70	54, 90	5, 31	40, 87	50, 111	13, 23	38, 48	51, 68
T2	5, 15	34, 47	45, 60	4, 18	27, 49	44, 64	1, 15	38, 49	39, 63
I2	3, 29	29, 50	48, 60	1, 41	15, 45	42, 58	(-6, 20)	40, 66	53, 69
Average Width	18.3	22.8	25.3	25	35	36.7	23.9	20.4	34.8

**Table 5.** Mean difference (limits of agreement) Before Correction. A: Before correction. B: After correction.

A. Mean difference (limits of agreement) before correction (µm)									
Subfield	All eyes			NV-AMD			NNV-AMD		
	Spectralis™ vs. Cirrus™	Cirrus™ vs. Stratus™	Spectralis™ vs. Stratus™	Spectralis™ vs. Cirrus™	Cirrus™ vs. Stratus™	Spectralis™ vs. Stratus™	Spectralis™ vs. Cirrus™	Cirrus™ vs. Stratus™	Spectralis™ vs. Stratus™
Central 1 mm	63 (228, -101)	52 (142, -38)	120 (305, -64)	64 (220, -92)	64 (165, -37)	127 (319, -64)	69 (244, -150)	32 (85, -21)	110 ( 287, -68)
N1	32 (112, -47)	50 (106, -6)	87 (168, 4)	35 (119, -49)	51 (115, -14)	92 (175, 8)	28 (85, -29)	49 (93, 7)	79 (158, 1)
S1	34 (108, -40)	58 (142, -26)	92 (203, -20)	40 (160, -80)	65 (164, -35)	103 (235, -28)	28 (68, -13)	47 (92, 1)	75 (134, 15)
T1	41 (143, -60)	55 (120, -10)	100 (217, -17)	52 (185, 82)	60 (138, 17)	107 (243, -27)	39 (129, -51)	47 (87, 7)	89 (172, 5)
I1	36 (100, -29)	58 (163, -47)	96 (195, -4)	32 (103, -38)	55 (182, -72)	94 (198, -10)	34 (100, -31)	62 (123, 1)	98 (194, 2)
N2	16 (40, -9)	42 (86, -2)	59 (98, 19)	14 (49, -19)	41 (95, -15)	59 (107, 11)	12 (34, -9)	44 (55, 23)	58 (81, 35)
S2	25 (74, -24)	58 (154, -38)	77 (175, -21)	23 (66, -20)	67 (186, -52)	88 (214, 40)	21 (38, 4)	44 (62, 25)	63 (93, 34)
T2	18 (50, -13)	51 (117, -15)	69 (145, -7)	20 (62, -22)	54 (132, -28)	70 (152, 13)	17 (41, -7)	46 (76, 15)	68 (138, -1)
I2	22 (95, -50)	40 (106, -26)	54 (147, -40)	26 (116, -62)	21 (104, -42)	56 (92, 19)	15 (58, -29)	53 (99, 7)	69 (105, -33)
B. Mean difference (limits of agreement) after correction (µm)									
Subfield	All eyes			NV-AMD			NNV-AMD		
	Spectralis™ vs. Cirrus™	Cirrus™ vs. Stratus™	Spectralis™ vs. Stratus™	Spectralis™ vs. Cirrus™	Cirrus™ vs. Stratus™	Spectralis™ vs. Stratus™	Spectralis™ vs. Cirrus™	Cirrus™ vs. Stratus™	Spectralis™ vs. Stratus™
Central 1 mm	19 (92, -54)	55 (150, -39)	73 (184, -38)	17 (80, -44)	70 (175, -34)	85 (204, 34)	20 (110, -69)	32 (85, -21)	52 (136, -33)
N1	12 (71, -47)	52 (95, 8)	67 (145, -12)	14 (79, -52)	53 (97, 9)	73 (154, -9)	10 (60, -39)	49 (93, 7)	56 (128, -16)
S1	17 (97, -62)	56 (139, -26)	72 (165, -21)	20 (116, -78)	62 (162, -36)	79 (185, -27)	15 (61, -31)	47 (92, 1)	60 (124, -4)
T1	23 (97, -51)	55 (116, -6)	76 (150, 1)	23 (71, -24)	60 (131, -11)	79 (143, 15)	23 (125, -79)	47 (87, 7)	70 (161, -21)
I1	11 (55, -33)	58 (161, -45)	73 (154, -7)	10 (59, -40)	55 (179, -68)	72 (150, -6)	13 (50, -24)	62 (123, 1)	75 (163, -12)
N2	10 (43, -23)	41 (83, -2)	53 (99, 7)	11 (47, -24)	39 (90, -13)	54 (103, 6)	8 (37, -21)	44 (55, 23)	51 (95, 9)
S2	19 (66, -29)	56 (148, -36)	73 (175, -30)	19 (79, -42)	64 (178, -50)	81 (210, -49)	18 (37, -1)	44 (62, 25)	60 (89, 31)
T2	14 (53, -33)	50 (115, -16)	64 (129, -1)	16 (66, -33)	52 (132, -28)	64 (131, 3)	12 (29, -5)	46 (76, 15)	64 (129, 0)
I2	16 (96, -64)	39 (104, -26)	54 (88, 20)	22 (115, -72)	31 (100, -39)	50 (86, 14)	7 (54, -34)	53 (99, 7)	62 (87, 37)

**Figure 2a–f.**
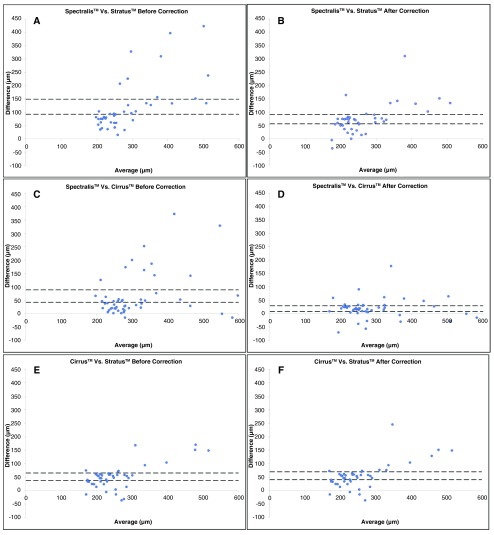
Bland-Altman Plots of agreement with 95% Confidence Intervals for the 1mm central subfield. **A**: Spectralis™ vs. Cirrus™ before correction. **B**: Spectralis™ vs. Cirrus™ after correction. **C**: Cirrus™ vs. Stratus™ before correction. **D**: Cirrus™ vs. Stratus™ after correction. **E**: Spectralis™ vs. Stratus™ before correction. **F**: Spectralis™ vs. Stratus™ after correction.

Figure 2a–f show Bland-Altman plots with 95% confidence intervals for the FTH comparison of the machines before and after correction. Before correction, the mean difference between the machines was 32µm for Spectralis™ vs. Cirrus™, 52µm for Cirrus™ vs. Stratus™, and 84µm for Spectralis™ vs. Stratus™. Manual correction reduced the differences, with it being 15µm for Spectralis™ vs. Cirrus™, 51µm for Cirrus™ vs. Stratus™, and 67µm for Spectralis™ vs. Stratus™. When stratified by diagnoses, the values were 34µm and 29µm for Spectralis™ vs. Cirrus™, 53µm and 47µm for Cirrus™ vs. Stratus™, and 88µm and 79µm for Spectralis™ vs. Stratus™ for NV-AMD and NNV-AMD before correction, respectively. After manual correction, the values reduced to 17µm and 14µm Spectralis™ vs. Cirrus™ and 70µm and 61µm Spectralis™ vs. Stratus™ for NV-AMD and NNV-AMD, respectively. The confidence interval widths, on average, were 5–10µm smaller than between an SD-OCT and TD-OCT machine. The average interval width decreased between 5–10µm after correction for any disease and comparison, except for the Cirrus™ vs. Stratus™ comparison.

## Discussion

The advent of OCT has revolutionized the way patients with retinal disorders are evaluated and monitored. However, like every new device, the current devices employing time- or spectral domain technology have certain limitations. One such common and clinically relevant issue is the presence of a random error in the identification of the inner and outer boundaries of the retina by the algorithm. With respect to AMD, studies have shown that in lesions such as fibrotic scars, choroidal neovascularization disrupting the RPE, and subretinal fluid the automated segmentation algorithms would produce errors because the software would not correctly delineate the outer retinal boundary ^3, 5^. In our study, we found that 66% of the Spectralis™, 14% of the Cirrus™ and 6.5% of the Stratus™ scans had algorithm errors. Giani *et al.* reported similar results; for Cirrus™, they reported 25% and 16% algorithm error rates for NNV-AMD and NV-AMD, respectively. However, for Spectralis™, they reported 16.67% and 57.6% algorithm error rates and 8.33% and 62.5% rates for Stratus™ for NNV-AMD and NV-AMD, respectively ^5^. Other studies have reported Stratus™ outer boundary algorithm errors of approximately 43% for both forms of AMD and 60% for NV-AMD ^3, 6^.

Reasons for differences in our error rates compared to previous include a lack of standard definition of an algorithm error. Rather than having an exact definition of an algorithm error, which may not be clinically significant ^5^, in our study, the decision was made by two masked observers who determined if the correction would be important. In addition, even though Spectralis™ segments the outer border of the RPE, a study by Jaffe *et al.* reported that it may also be including the Bruch’s membrane in its calculation, thus including sub-RPE pathology such as drusen when segmenting the outer border of the retina ^7^. These differences may be due to the fact that our study was prospective and while acquiring scans, the operators tried their best to ensure no errors occurred during scan acquisition. Lastly, we did not exclude scans if the signal strength was low or if the machine gave a low analysis confidence message, as other studies have done ^8– 10^.

After correction the thickness measurements for the Spectralis™ and Cirrus™ scans were not significantly different. This may be due to the fact that the majority of the scans required minor corrections. For example, more than 50% of the Spectralis™ scans resulted in a 10µm or less change in the central subfield thickness. Krebs *et al.* have also previously reported no significant differences in retinal thickness measurements before and after correction of segmentation errors of scans taken using Cirrus™ ^11^.

The differences in the mean thickness values before and after correction in scans taken using Spectralis™ were most obvious in the central subfields of the retina (C1, N1, S1, T1, and I1) with the peripheral subfields being spared (N2, S2, T2 and I2). This may be attributed to the fact that the pathology of AMD is located centrally and therefore pathology related inaccuracies in segmentation are more likely to occur in these subfields.

Retinal thickness measurements were similar in both SD-OCT machines and were greater than Stratus™. Correction reduced the difference of the thickness measurements between the two SD-OCT devices to less than 20um; in some cases as noted above, the difference was no longer statistically significant. Other studies in normal and pathologic eyes including DME and macular degeneration have also demonstrated that the difference in retinal thickness between the SD machines can be attributed to the differences in segmentation of the automated algorithms ^7, 10, 12^.

Despite the large numbers of scans with algorithm errors, the COR of Spectralis™ was lower for every subfield than that of Stratus™ or Cirrus™. The COR of Cirrus™ was equal to or larger than Stratus™ for both forms of the disease. In all three devices, the COR was generally better for NNV-AMD when compared to NV-AMD, especially after correction. The disease difference can be attributed to the pathology of NV-AMD disrupting the outer border, which makes it difficult for the automated algorithm to accurately segment the retinal layers ^13, 14^. Krebs *et al.* evaluated the repeatability of retinal thickness measurements using Spectralis™ and Cirrus™ in patients with AMD. For images taken using Spectralis™ the mean difference between repeated measurements was found to be within 11µm before correction and within 1µm after correction. For images taken using Cirrus™ the mean difference between repeated measurements was found to be within 6µm before correction and within 4µm after correction ^15^. Previous studies on normal eyes have reported a high repeatability of measurements with Spectralis™, with differences between repeated measurements being within 1µm ^12, 16^. For Stratus™ OCT images, other studies have found central subfield repeatability values in patients with NV-AMD to be 50µm and 32–35µm for NNV-AMD patients after correction/exclusion of scans with errors ^8, 17^; our study confirms this finding. There has been one other published study looking at the repeatability of Cirrus™ OCT in NV-AMD, which found a central subfield repeatability value of 42um before correction and 26µm after exclusion of scans with significant segmentation errors ^18^. The difference between this study and our measurements may be associated with our smaller sample size. In addition, we chose not to exclude any poor quality scans, which may cause larger differences.

In addition to a lower COR, Spectralis™ also had the highest ICC values for both NV-AMD and NNV-AMD, before and after correction. For NV-AMD, Cirrus had higher coefficients after correction, and for NNV-AMD, Cirrus™ had lower coefficients as compared to Stratus™. While no previous studies have reported ICC values for AMD patients, Pierro *et al.* found comparable results in normal eyes, with Cirrus™ ICC values ranging from 83–97% and Stratus™ ICC values from 72–95% ^19^. The most likely reason for the low repeatability and high ICC values for Spectralis™ is the eye-tracking capability, which ensures that artifacts due to eye movement are minimized and the machine scans only when the tracking software identifies the same position on the fundus ^16^.

Bland-Altman plots indicate that there is agreement between SD-OCT machines. Correcting images also influenced agreement between machines. We found that 95% confidence intervals were narrower as compared to an SD-OCT and TD-OCT and correcting the algorithm errors further narrowed the intervals. The mean difference between machines indicates that the lowest differences were between Spectralis™ and Cirrus™, especially after correction. This is mostly likely due to the effects of manually correcting the Spectralis™ images and that both machines have similar scanning technologies. The limits of agreement were similarly very wide for all three machines, and were narrower after correction of images, especially for the two SD-OCT machines. Jaffe *et al.* reported similar results looking at NV-AMD, with limits of agreements being approximately 225um between a SD-OCT and TD-OCT ^7^. The poor agreement warrants caution for clinicians when trying to use the data from different machines interchangeably especially in the central 1mm of retina since most clinicians.

Our study is not without its limitations. All images were taken at a single imaging center; this might have introduced some bias. The version of software used for the Stratus™ images did not allow correction of segmentation errors and therefore these images had to be excluded from the analysis. Two independent graders manually corrected all the images; this may have resulted in some inaccuracies in segmentation line correction. In addition, in a subset of patients that had a difference in the severity of disease, both eyes were included in the analysis; this may also have resulted in possible bias. The Cirrus device that was used to capture the images did not have eye tracking and may have led to the slightly larger COR values when compared to Spectralis.

In summary, we found that although Spectralis™ had the highest frequency of errors in AMD patients, correction of images did not result in significant changes in retinal thickness due to the errors being very small. Spectralis™ had the lowest COR values. Thus Spectralis™ maybe the best suited for examining minute morphological and thickness changes. Also, because of the wide Bland-Altman 95% intervals, there is not much agreement between the SD-OCT and TD-OCT machines. Based on our findings, we recommend that scans be carefully analyzed at reading centers before the thickness values are accepted as reliable.

## Figures and Tables

**Figure:**
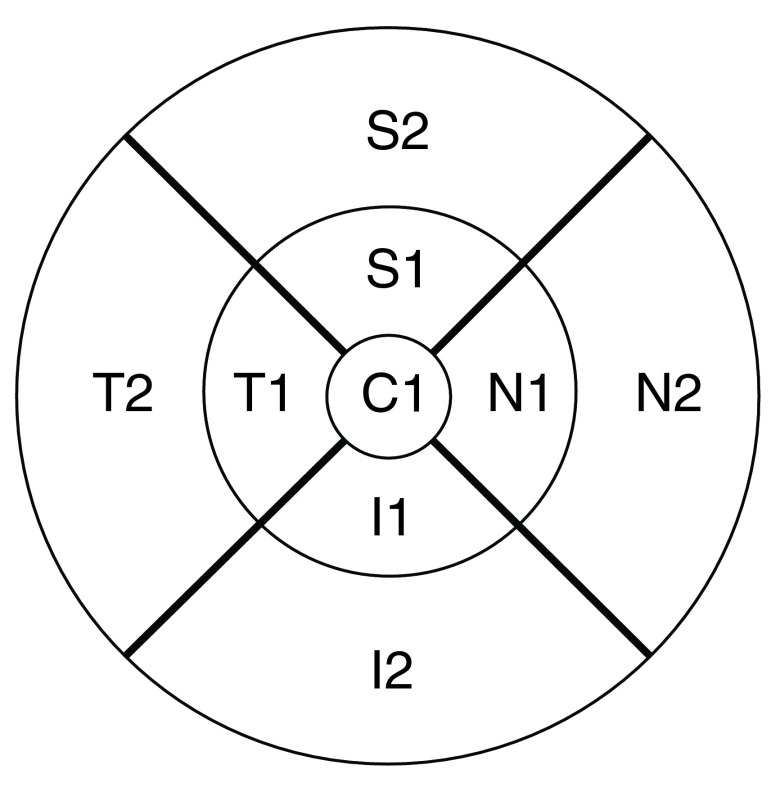


## Appendix A

The following ETDRS grid depicts the abbreviations for the nine subfields. C1 – Central 1mm. N1 – Inner nasal. S1 – Inner superior. T1 – Inner temporal. I1 – Inner inferior. N2 – Outer nasal. S2 – Outer superior. T2 – Outer temporal. I2 – Outer inferior.
