# Supplementary material for: Reliability and reproducibility of spectral and time domain optical coherence tomography images before and after correction for patients with age-related macular degeneration
Source: F1000Res. 2015 Mar 5;2:131. Originally published 2013 May 23. [Version 2] doi: 10.12688/f1000research.2-131.v2 (PMC4457115; doi:10.12688/f1000research.2-131.v2)
Supplement: Peer review history

# Peer review: Referee response for version 2

**DOI:** 10.5256/f1000research.6502.r8817

**Published:** 2015-06-03

**Authors:** Akshay Nair

**Affiliations:** 1 Department of Neuro-Opthalmology, Sankara Nethralaya, Chennai, India

This study was performed to assess the reproducibility and reliability of optical coherence tomography scans obtained using the time domain (TD-OCT) Stratus OCT, and the Spectral Domain (SD-OCT) Spectralis and Cirrus OCT devices before and after manual correction. 

The title and abstract are appropriate.  The possible errors and drawbacks have been addressed and mentioned in the manuscript.  The statistical analysis is appropriate and the authors have presented acceptable conclusions. The conclusions are are acceptable and balanced; however, the number of eyes included in the study could have been more. The previous reviewers have addressed important point which have been duly taken into consideration by the authors.

I have read this submission. I believe that I have an appropriate level of expertise to confirm that it is of an acceptable scientific standard.

# Peer review: Referee response for version 2

**DOI:** 10.5256/f1000research.6502.r7925

**Published:** 2015-03-17

**Authors:** Igor Kozak

**Affiliations:** 1 Vitreoretinal Division, King Khaled Eye Specialist Hospital, Riyadh, Saudi Arabia

The authors have addressed all reviewers comments.

I have read this submission. I believe that I have an appropriate level of expertise to confirm that it is of an acceptable scientific standard.

# Peer review: Referee response for version 1

**DOI:** 10.5256/f1000research.1263.r4688

**Published:** 2014-05-12

**Authors:** Igor Kozak

**Affiliations:** 1 Vitreoretinal Division, King Khaled Eye Specialist Hospital, Riyadh, Saudi Arabia

This study reports on reliability and reproducibility of optical coherence tomography (OCT) scans before and after manual correction in eyes with age-related macular degeneration (AMD). It concludes that manual correction improves automated segmentation and that the agreement, as evidenced by intraclass correlation coefficients and coefficients of repeatability, is better between spectral-domain OCT instruments. The study brings important numerical comparisons of measurements of central foveal thickness using different instruments. Such comparisons are crucial especially with transitioning from time-domain to spectral-domain OCT technology in ongoing and future clinical trials. The paper is well written. Minor comments for authors:

1. The same operator acquired OCT scans of the same eyes on the same instruments. It would be useful to know how many graders and how independently performed manual correction at the Reading Center.
2. The Spectralis system via TruTrack provides excellent ability to perform follow-up scans from the exact retinal areas. How was this dealt with using other two systems in order to avoid sampling error?
3. Misidentification of inner retinal later is a common artifact and has been found in a large number of scans including the instruments used in this study. The authors are encouraged to cite some of those studies such as Ho *et al. *(2009).
4. Another useful study to mention with respect to comparing time-domain and spectral-domain OCT instruments: Mylonas *et al. *(2009).
5. Based on a study of reproducibility in Stratus OCT, any artifact resulting in an error that is more than 50μm is clinically significant, suggesting 50 μm as a cutoff for retreatment of neovascular AMD patients (Patel *et al., *2009). In another study any artifacts resulting in automated segmentation errors of more than 10% of the actual (manually measured) ETDRS center subfield thickness were considered clinically significant ( Browning *et al.*, 2008). In this study, some of the variations after manual correction surpassed these margins.  Maybe some comment in Discussion regarding this issue.

I have read this submission. I believe that I have an appropriate level of expertise to confirm that it is of an acceptable scientific standard.

# Peer review: Referee response for version 1

**DOI:** 10.5256/f1000research.1263.r3258

**Published:** 2014-02-03

**Authors:** Ilse Krebs

**Affiliations:** 1 Department of Ophthalmology, Rudolf Foundation Clinic, Vienna, Austria

The reproducibility of retinal thickness measurements with different OCT devices in eyes with age-related macular degeneration before and after error correction is presented. This is a topic of high interest and actuality. The title and Abstract are appropriate.

- *"To date, no other study has examined the effects of manual correction of the thickness algorithm in SD-OCT and TD-OCT machines in eyes with AMD."*
- *"At this point, we are not aware of any previous study looking at the repeatability of Spectralis™ images in AMD."*

This is not really true, and I want to refer to publications dealing with this topic ( Krebs *et al. *2012; Krebs *et al.,* 2011; Krebs *et al.*, 2009). The results of these studies should be discussed, as some of the results are confirmed by the results of the current study. Most of the studies focus only on the central 1000µm area, whereas in this study also more peripheral areas were examined. This should be discussed a little bit more because this might be interesting: were the failures only in the central part, or also in the periphery (the pathology of neovascular AMD is located centrally therefore pathology related failures should occur only in the central area.) 

- It is mentioned in the discussion section, but it should also added to the methods: how many observers assessed the segmentation errors, and performed the error correction?
- The lack of significant differences before and after correction might be due to the small number of examinations requiring correction.
- The sample size seems to be quite low, was there any calculation when planning this study? A possible bias of including both eyes in a part of patients should be mentioned.

I have read this submission. I believe that I have an appropriate level of expertise to confirm that it is of an acceptable scientific standard, however I have significant reservations, as outlined above.

# Response

**Published:** 2014-02-28

**Authors:** Yasir Sepah

**Affiliations:** Department of Ophthalmology and Visual Sciences, University of Nebraska Medical Center, USA

Dear Dr. Krebs,

Thank you for your valuable comments. We agree that our study is not the only study that has tried to deal with this topic. We will make changes to the manuscript to clarify this statement.

We will add the results and analysis of the peripheral areas to the discussion in the revision.

No sample size calculation was performed before the conduct of the study. Two eyes of the same patient were included because of difference in the severity of the disease between the two eyes of the same patient. The control group patients contributed only one eye to the analysis.
